# Establishment of an unfed strain of *Paramecium bursaria* and analysis of associated bacterial communities controlling its proliferation

**DOI:** 10.3389/fmicb.2023.1036372

**Published:** 2023-03-07

**Authors:** Eiko Himi, Tohru Miyoshi-Akiyama, Yuri Matsushima, Iru Shiono, Seiji Aragane, Yui Hirano, Gaku Ikeda, Yuki Kitaura, Kyohei Kobayashi, Daichi Konno, Ayata Morohashi, Yui Noguchi, Yuka Ominato, Soma Shinbo, Naruya Suzuki, Kurama Takatsuka, Hitomi Tashiro, Yoki Yamada, Kenya Yamashita, Natsumi Yoshino, Masaharu Kitashima, Susumu Kotani, Kazuhito Inoue, Akiya Hino, Hiroshi Hosoya

**Affiliations:** ^1^Faculty of Agriculture, Kibi International University, Minamiawaji, Hyogo, Japan; ^2^Department of Infectious Diseases, Research Institute, National Center for Global Health and Medicine, Tokyo, Japan; ^3^Department of Biological Sciences, Graduate School of Science, Kanagawa University, Kanagawa, Japan; ^4^Department of Biological Sciences, Faculty of Science, Kanagawa University, Kanagawa, Japan; ^5^Research Institute for Integrated Science, Kanagawa University, Kanagawa, Japan

**Keywords:** *Paramecium bursaria*, symbiosis, symbiotic algae, bacteria, unfed strain, protist, unfed culture, lettuce medium

## Abstract

The ciliate *Paramecium bursaria* harbors several hundred symbiotic algae in its cell and is widely used as an experimental model for studying symbiosis between eukaryotic cells. Currently, various types of bacteria and eukaryotic microorganisms are used as food for culturing *P. bursaria*; thus, the cultivation conditions are not uniform among researchers. To unify cultivation conditions, we established cloned, unfed strains that can be cultured using only sterile medium without exogenous food. The proliferation of these unfed strains was suppressed in the presence of antibiotics, suggesting that bacteria are required for the proliferation of the unfed strains. Indeed, several kinds of bacteria, such as *Burkholderiales*, *Rhizobiales*, *Rhodospirillales*, and *Sphingomonadales*, which are able to fix atmospheric nitrogen and/or degrade chemical pollutants, were detected in the unfed strains. The genetic background of the individually cloned, unfed strains were the same, but the proliferation curves of the individual *P. bursaria* strains were very diverse. Therefore, we selected multiple actively and poorly proliferating individual strains and compared the bacterial composition among the individual strains using 16S rDNA sequencing. The results showed that the bacterial composition among actively proliferating *P. bursaria* strains was highly homologous but different to poorly proliferating strains. Using unfed strains, the cultivation conditions applied in different laboratories can be unified, and symbiosis research on *P. bursaria* will make great progress.

## Introduction

The unicellular ciliated protist *Paramecium bursaria* has hundreds of symbiotic algae within its cell. Numerous studies have revealed that *P. bursaria* can proliferate not only by consuming surrounding microorganisms such as bacteria, but also by utilizing the photosynthetic products produced by their symbiotic algae (Siegel, 1960; Karakashian, 1963; Reisser, 1988).

Since both *P. bursaria* and symbiotic algae are eukaryotic unicellular organisms, *P. bursaria* is considered a suitable model ciliate for investigating the symbiotic association between eukaryotic cells (Margulis and Bermudes, 1985; Fujishima, 2009). Since *P. bursaria* is easy to cultivate, many research results regarding *P. bursaria* have been published. However, *P. bursaria* cultivation conditions are not the same among laboratories, which has a significant impact on the reproducibility of experiments in the field.

The green *P. bursaria* is most commonly cultivated in lettuce media containing exogenous microorganisms provided as food (Barna and Weis, 1973; Weis, 1975). The type of food varies depending on laboratories and research groups, and a wide variety of microorganisms, such as *Klebsiella pneumoniae* (Berk et al., 1991; Kosaka, 1991; Greczek-Stachura et al., 2021a) and *Chlorogonium elongatum* (Sonneborn, 1970; Steinbrück et al., 1981; Görtz, 1982; Omura et al., 2004), are used. However, the ingredients of lettuce leaves differ from region to region (Barta and Tibbitts, 1991; Boroujerdnia and Ansari, 2007; Urbonavičiūtė et al., 2007; Chang et al., 2014), and cultivation conditions are not standardized. We believe that unifying cultivation conditions is an urgent task and will improve the reproducibility of experiments using *P. bursaria*.

Previous reports have stated that inorganic salt solutions could be used for culturing ciliates (Raffel, 1930; Kirby, 1950), such as the commonly applied KNOP solution, modified Bold’s Basal Medium, etc. (Omura et al., 2004; Spanner et al., 2022). However, a detailed analysis has not yet been performed to determine the extent to which inorganic salt solutions are suitable for culturing *P. bursaria*. The development of a *P. bursaria* culture medium with a constant composition to replace the lettuce medium is important.

There are problems that need to be solved to unify the food provided for *P. bursaria*. First, the purity of the food needs to be checked. For example, when using a specific microbe as food, it should not be contaminated with other microbes. However, it is not easy to establish an axenic culture system for food microbes, and it is not clear whether this is possible. However, it is necessary to determine the most suitable microbe for culturing *P. bursaria* (Bator, 2010) and share the microbes with *P. bursaria* researchers worldwide. However, because the diversity of *P. bursaria* used by each researcher is unknown, it is unrealistic to determine the best food. Rather, it is more realistic to establish a new *P. bursaria* strain (unfed culture strain) that can be cultured without feeding.

It has long been known that *P. bursaria* is able to use the photosynthetic products obtained from hundreds of symbiotic algae in the cell (Brown and Nielsen, 1974; Reisser, 1976). Therefore, *P. bursaria*, unlike *P. caudatum* and other species of paramecia, have the ability to survive using only photosynthetic products without requiring exogenous microbes. If an unfed culture strain could be established, it would be possible to share and use this strain widely among researchers, which would solve the food inconsistency problem. This would greatly advance the unification of *P. bursaria* cultivation conditions among researchers.

In this study, in 2015, we collected *P. bursaria* from a pond on our campus and cloned it twice (Hosoya et al., 2017). After cultivating *P. bursaria* as fed cultures with exogenous bacteria for 1 year, we continued cultivation using sterile lettuce medium without any exogenous bacteria as food (unfed culture). In 2017, to initiate unfed cultures, we isolated *P. bursaria* individuals again from the fed culture strain (third cloning), washed them four times by allowing them to swim in sterile lettuce media, and placed individually washed *P. bursaria* into sterile lettuce medium. We were able to maintain the unfed culture strains until 2022 using only sterile lettuce medium, without administering any exogenous food. By sharing the unfed culture strains established here with researchers from the *P. bursaria* field, we believe that we could greatly contribute to the unification of *P. bursaria* cultivation conditions.

Interestingly, bacteria were always observed in the medium of the unfed culture strains, even after long-term cultivation in sterilized lettuce media. Unfed culture strains did not proliferate in the presence of antibiotics, suggesting that bacteria are essential for the proliferation of *P. bursaria*. 16S rDNA sequencing analysis showed that multiple species of bacteria with bioremediation ability and plant proliferation-promoting activity were detected in unfed culture strains. In this paper, we report our results on the role of these bacteria in the proliferation of *P. bursaria*.

## Materials and methods

### Isolation of a *Paramecium bursaria* strain and its accompanying bacteria

**Figure 1:**
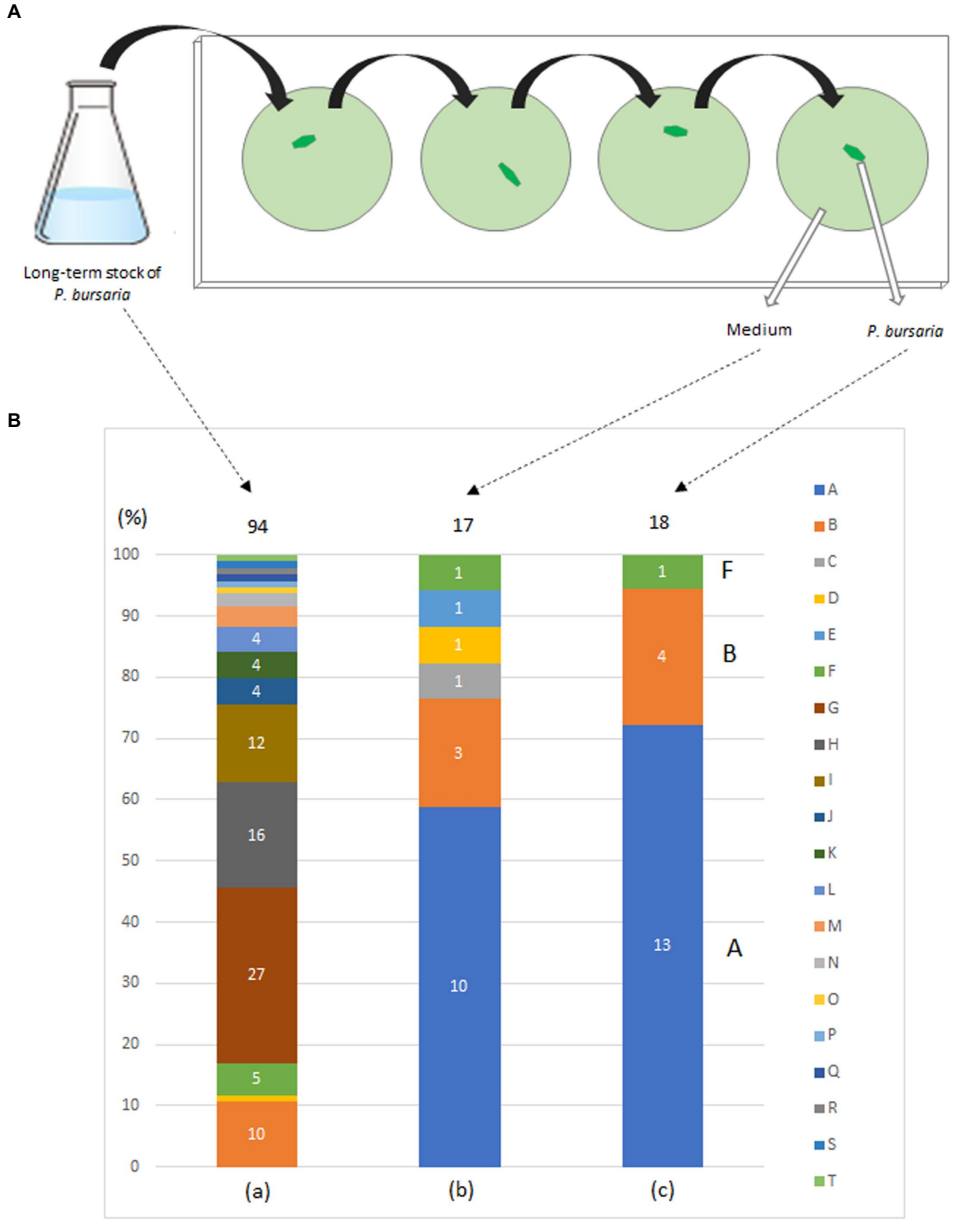
Washing procedure **(A)** and bacterial taxa **(B)** detected in a fed culture strain of *P. bursaria* (KUNY-2). **(A)** A single *P. bursaria* cell isolated from a fed culture was allowed to swim (1–2 min) in a drop of sterile lettuce medium to wash it from other microbes present. Then, the individual was recovered, placed in a new fresh drop of sterile lettuce medium and allowed to swim to wash it again. The same washing procedure was repeated two more times, and finally the *P. bursaria* individual and the medium were separately placed and spread on BTB agar plates to grow bacterial colonies. **(B)** (a) Bacterial colonies in the medium (94 colonies), (b) in the medium after washing the ciliate 4 times (17 colonies), and, (c) in one washed *P. bursaria* cell (18 colonies). Identified bacteria are color-coded (A–T). Numbers in bar graphs indicate the number of identified colonies per taxon. In (a), when the number of colonies is 3 or less, it is not shown in the bar graph. In (c), A, B, and F indicate bacteria corresponding to A, B, and F among the bacteria identified below. A, *Caulobacter* sp.; B, *Variovorax paradoxus*; C, *Chryseobacterium gambrini*; D, *Novosphingobium* sp.; E, *Paracoccus yeei*; F, *Variovorax* sp.; G, *Ancylobacter* sp.; H, *Aquabacter spiritensis*; I, *Kaistia* sp.; J, *Caulobacter* endosymbiont of *Tetranychus urticae*; K, *Dyadobacter jiangsuensis*; L, *Elizabethkingia* sp.; M, *Pelomonas* sp.; N, *Microbacterium* sp.; O, *Leifsonia* sp.; P, *Microbacterium paraoxydans*; Q, *Nevskia* sp.; R, *Pseudoxanthobacter liyangensis*; S, *Sphingomonas* sp.; T, *Xanthobacter flavus.*

In May 2015, *P. bursaria* was collected from a pond on the campus of Kanagawa University, Kanagawa Prefecture, Japan, and a fed culture of *P. bursaria* was initiated in a lettuce medium to which the bacterium *Klebsiella* sp. (JCM14683, RIKEN, Japan) (Elbeltagy et al., 2000) was added. Bacterized medium was prepared for each experiment. In June, *P. bursaria* individuals were isolated and cloned from the culture medium, washed four times with sterile lettuce medium as shown in Figure 1A, and the fed culture of the washed individuals was resumed in a 6-well plate (TPP 92006, flat bottom; 3 ml/well; see (Matsushima et al., 2022) for details on washing methods). Single *P. bursaria* were isolated from the culture again in July, washed four times with sterile lettuce medium as described above, and then subjected to a second cloning and washing. A fed culture of the individuals was reinitiated in a 6-well plate (strain KUNY-2). In August, each entire culture in the well was transferred into 150 mL of lettuce medium containing originally accompanying bacteria in a 200 mL flask (Pyrex, Iwaki Glass, Japan), and a large-volume culture was started. The culture was continued in an incubator (Biotron, LH-411S) at 23°C under light irradiation (12 h light/12 h dark, 400 μmol photon·m^−2^·s^−1^), and lettuce medium with bacteria was added to the flasks in August 2016, about 1 year later. The culture was continued from October to November, and the culture medium was plated on bromothymol blue (BTB) agar (Kyokuto Pharmaceutical, Japan) after dilution. Ninety-four bacterial colonies were isolated, and the bacteria were identified by 16S rDNA sequencing using the conventional Sanger method and BLAST analysis with the 16S rDNA sequence database (for Figure 1). At the same time, individual *P. bursaria* were isolated from the culture medium and washed four times with sterile lettuce medium. Subsequently, the washed individual *P. bursaria* and the last washing medium were separately applied to BTB agar plates. The bacterial colonies obtained (18 for the former and 17 for the latter) were identified using the method described above.

### Establishment of unfed culture strains of *Paramecium bursaria*

The unfed culture for the KUNY-2 strain was initiated in March 2017. *Paramecium bursaria* individuals were isolated from a large-volume culture (200 mL flask) and washed four times with sterile lettuce medium (third cloning). Individually washed *P. bursaria* were cultured separately in four 6-well plates (3 mL/well) in sterile lettuce medium. By June, five unfed culture strains with a high proliferation density were selected from each of the 6-well plates and named as KUHH-1, 3, 4, 6, and KUYY strains.

### Cultivation in the presence of antibiotics

The KUYY strain was cultured in sterile lettuce culture medium (6-well plate, 3 mL/well) with or without various concentrations of antibiotics. Proliferation was monitored over time by directly counting the number of paramecia in the culture, using an inverted microscope. Penicillin (10,000 U/mL)/Streptomycin (10,000 μg/mL) mixture (168–23,191, Wako/Fujifilm, Japan) at five concentrations ranging from 10^−6^ to 10^−2^ and Gentamicin (073–02971, Wako/Fujifilm, Japan) at four concentrations ranging from 0.01 to 10 μg/mL were applied to the KUYY strain (around 21–36 cells/mL) and the changes in cell density were measured for 2–3 weeks. Proliferation was measured in duplicates at each concentration, and the standard deviation calculated.

### Proliferation of unfed *Paramecium bursaria* strains

**Figure 2:**
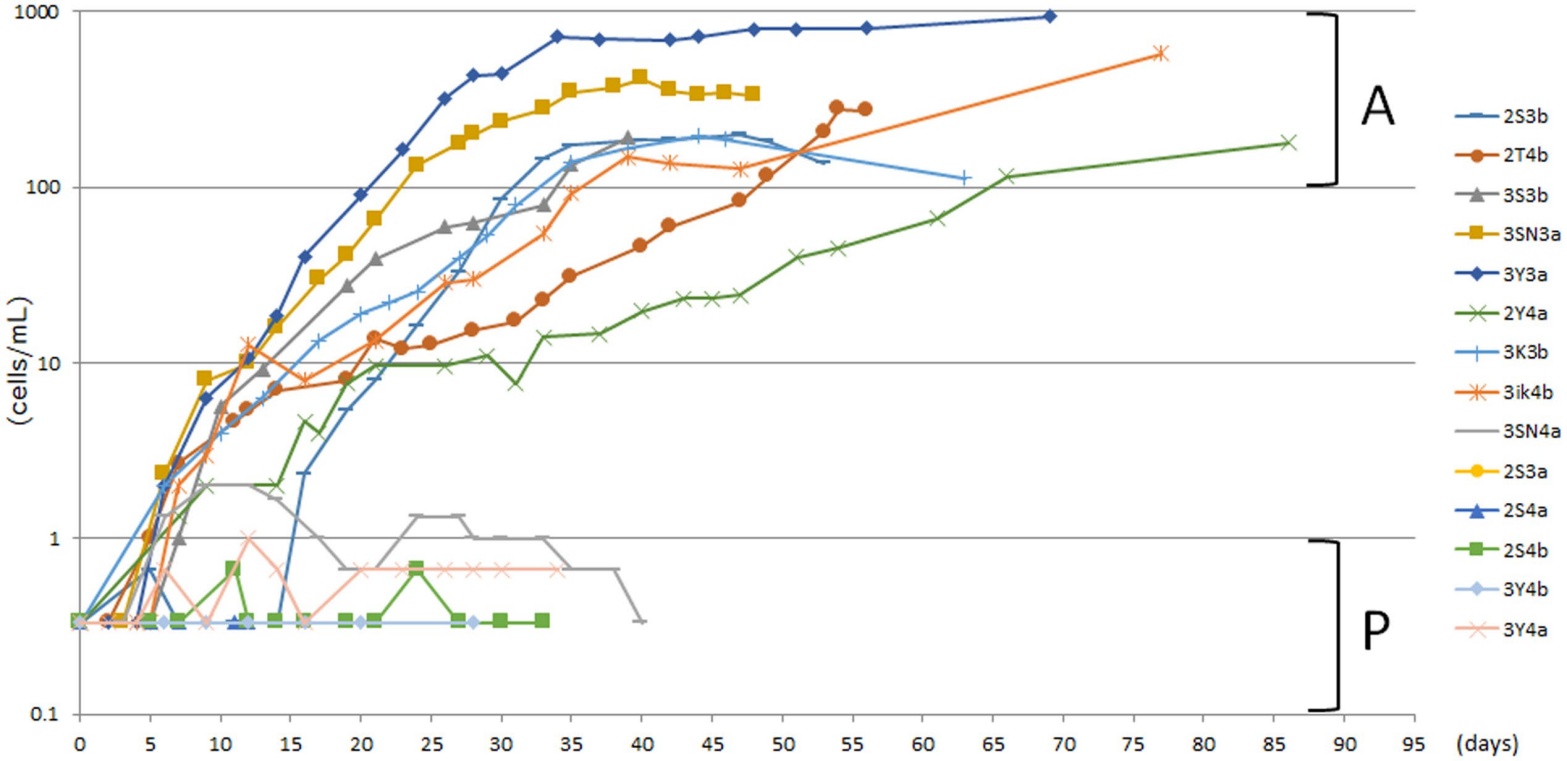
Actively and poorly proliferating strains in unfed cultures of *P. bursaria*. Strains with a stationary phase cell density of 100 cells/mL or more were defined as actively proliferating strains (A), and those with a stationary phase cell density of 1 cell/mL or less were defined as poorly proliferating strains (P). Among these strains, eight and six representative strains were selected as A and P, respectively. The names of each of the 14 strains are shown on the right-hand side. The top eight and the bottom six listed vertically in the figure correspond to strains A and P, respectively. The abscissa represents the number of days from the start of culture and the ordinate represents the cell density.

The unfed culture strains (KUHH-3 and 4), which were cloned in 2017 for the third time and then cultured under unfed cultivation conditions, were cloned again (for the fourth time in total) in 2020 and proliferation was monitored over time. For Supplementary Figures S1D,E, freshly prepared dried lettuce leaves were used to prepare lettuce medium. In all five experiments (Supplementary Figures S1A–E), individual *P. bursaria* were cloned from the KUHH-3 and 4 stocks. Based on the cell density in the stationary phase, each strain was classified into four stages: 100–1,000 cells/mL (actively proliferating strains, denoted as A), 10–99 cells/mL, 1–9 cells/mL, and less than 1 cell/mL (poorly proliferating strains, denoted as P). From three out of five experiments shown in Supplementary Figure S1, eight strains with active proliferation (A) and six strains with poor proliferation (P) were selected (Figure 2). Next, for these 14 unfed strains of *P. bursaria*, we analyzed the microbiome composition in *P. bursaria* cells and in the medium.

### Microbiome analysis by 16S rDNA sequencing

The *P. bursaria* culture was centrifuged at 150 *× g* for 30 min, and the cells were recovered as a precipitate. The supernatant was further centrifuged at 4,500 *× g* for 5 min to collect bacteria. DNA was isolated separately from *P. bursaria* and bacterial fractions, and PCR was performed using a probe containing the bacterial-specific V3/V4 region sequence of the 16S rRNA gene. The V3/V4 region sequences of the 16S rRNA gene were amplified using primers with adapter sequences 341f: 5′-ACACTCTTTCCCTACACGACGCTCTTCCGATCT-NNNNN-CCTACGGGNGGCWGCAG-3′ and 805r: 5′-GTGACTGGAGTTCAGACGTGTGCTCTTCCGATCT-NNNNN-GACTACHVGGGTATCTAATCC-3′.^1^ The adaptor and barcode sequences were appended using a second round of PCR. The second round of PCR and amplicon sequencing were performed on a MiSeq system (Illumina) and 300 bp paired-end reads were sequenced to identify individual bacteria and their composition. The amplicon sequence variant (ASV) abundance of each of the identified bacteria was determined among eight *P. bursaria* strains with active proliferation (28 combinations in total), six strains with poor proliferation (15 combinations in total), and between combinations of the two from all strains (48 combinations in total). ASV clustering and differential abundance analyses were performed using the QIIME2 pipeline (ver. 2022.2).^2^ To identify the bacteria, the Greengenes (ver. 13_8) database was used, based on 97% operational taxonomic unit (OTU) clustering thresholds in the module. In addition, Linear discriminant analysis Effect Size (LEfSe) analysis was performed to clarify the similarity in the bacterial composition of each *P. bursaria* strain based on the calculation of the correlation coefficient.^3^ Analyses were performed using multiple comparison analysis with Bonferroni correction in R (ver. 4.1.2). The data presented in this study are deposited in the repository of the DNA Data Bank of Japan (DDBJ) (DDBJ accession numbers DRA014782 and LC726600-LC726729). All analyses were conducted at the Bioengineering Lab. Co. Ltd. (Sagamihara, Kanagawa, Japan). The bacterial abundance analysis was performed using Microbial genomics module in CLC genomics workbench (ver 11.0.1) (QIAGEN).

## Results

### Bacteria identified in washed and fed *Paramecium bursaria*

Figure 1B shows the bacterial composition identified from each individual *P. bursaria* isolated from the culture and washed four times with sterile lettuce medium (right bar) and from the media of the fed culture (KUNY-2; left bar). The central bar indicates the bacteria present in the medium after the fourth washing. Twenty taxa of bacteria, i.e., *Ancylobacter* sp., *Aquabacter spiritensis*, *Caulobacter* endosymbiont of *Tetranychus urticae*, *Caulobacter* sp., *Chryseobacterium gambrini*, *Dyadobacter jiangsuensis*, *Elizabethkingia* sp., *Kaistia* sp., *Leifsonia* sp., *Microbacterium paraoxydans*, *Microbacterium* sp., *Nevskia* sp., *Novosphingobium* sp., *Paracoccus yeei*, *Pelomonas* sp., *Pseudoxanthobacter liyangensis*, *Sphingomonas* sp., *Variovorax paradoxus*, *Variovorax* sp., and *Xanthobacter flavus* were detected. As can be seen in Figure 1B, 20 taxa of bacteria were detected from 94 colonies in the stock (a), whereas only three taxa of bacteria were detected from 18 colonies in the washed *P. bursaria* individuals (c). Notably, *Klebsiella* sp. used in fed cultures was not detected in any of the samples.

### *Paramecium bursaria* proliferation in the presence of antibiotics

**Figure 3:**
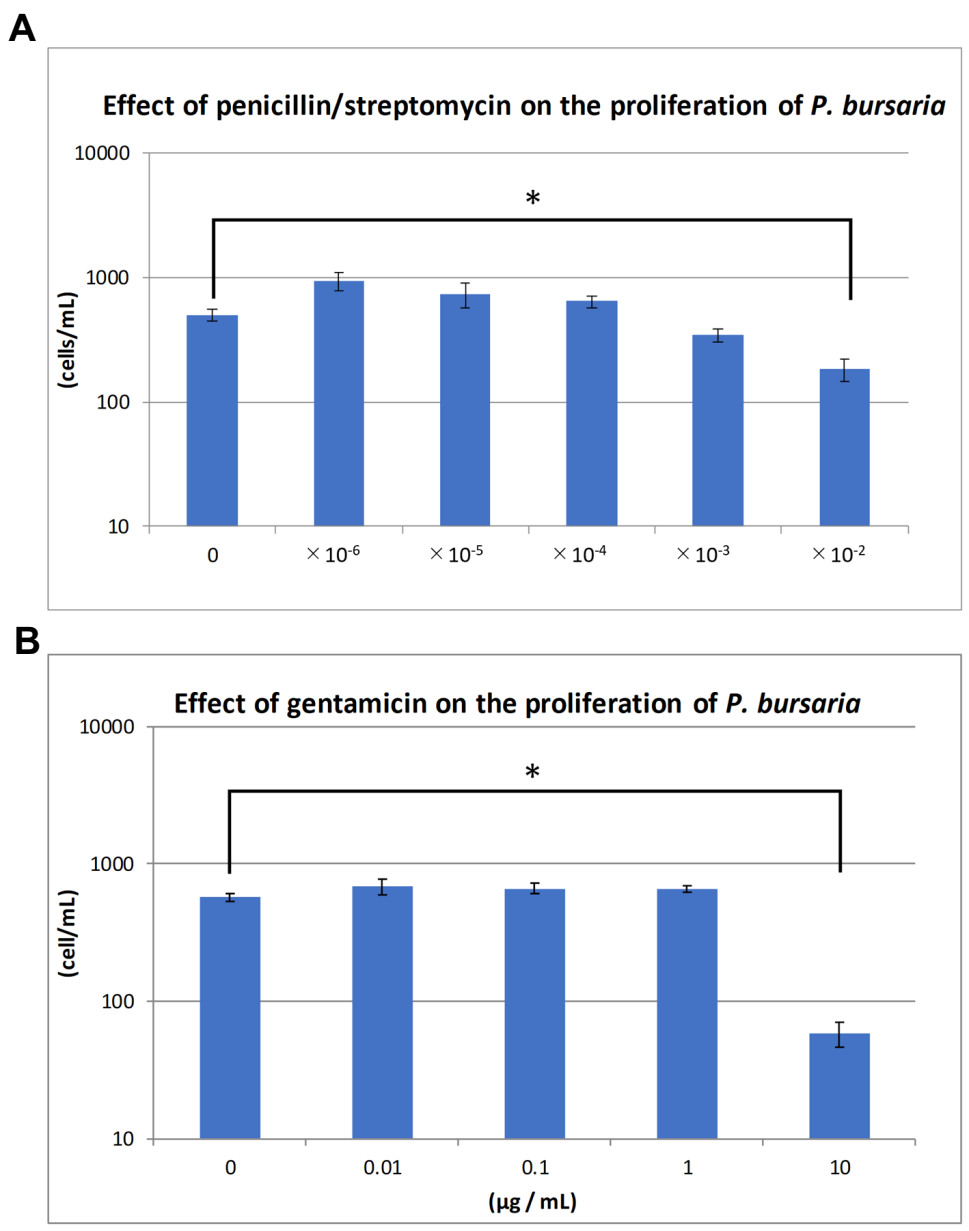
Effects of antibiotics on the proliferation of unfed culture strains. The abscissa indicates the concentrations of the penicillin/streptomycin mixture and gentamicin for **(A)** and **(B)**, respectively. The ordinate in **(A)** and **(B)** is the cell density (cells/mL) at 19 and 14 days after the start of culture, respectively. Asterisks indicate that differences are statistically significant from each other.

Figures 3A,B show the degree of *P. bursaria* proliferation after the addition of penicillin/streptomycin and gentamicin, respectively. The asterisk in the Figure 3 indicates that the proliferation of *P. bursaria* was significantly suppressed by the highest concentration of antibiotics used in this experiment. In the presence of gentamicin (100 μg/mL), many *P. bursaria* were distorted (data not shown).

### Proliferation of *Paramecium bursaria* strains after long-term unfed culture

Proliferation of the unfed strains (KUHH-3 and 4) was monitored over time for a large number of cloned strains by 10 researchers (Supplementary Figure S1). The three panels on the left (Supplementary Figures S1A,B,D) show the proliferation of single *P. bursaria* individuals isolated on three different occasions (October, November, and December 2020) from KUHH-3. The two panels on the right (Supplementary Figures S1C,E) show the proliferation curves of individuals isolated from KUHH-4 (November and December 2020). Each graph shows the proliferation of 20 strains (2 strains per each researcher). As shown in Supplementary Figure S1, the degree of proliferation was not affected by the freshness of the lettuce medium, by the original strain being KUHH-3 or KUHH-4, or by the researcher’s isolation technique.

### Analysis of bacteria associated with *Paramecium bursaria* by 16S rDNA sequencing

**Figure 4:**
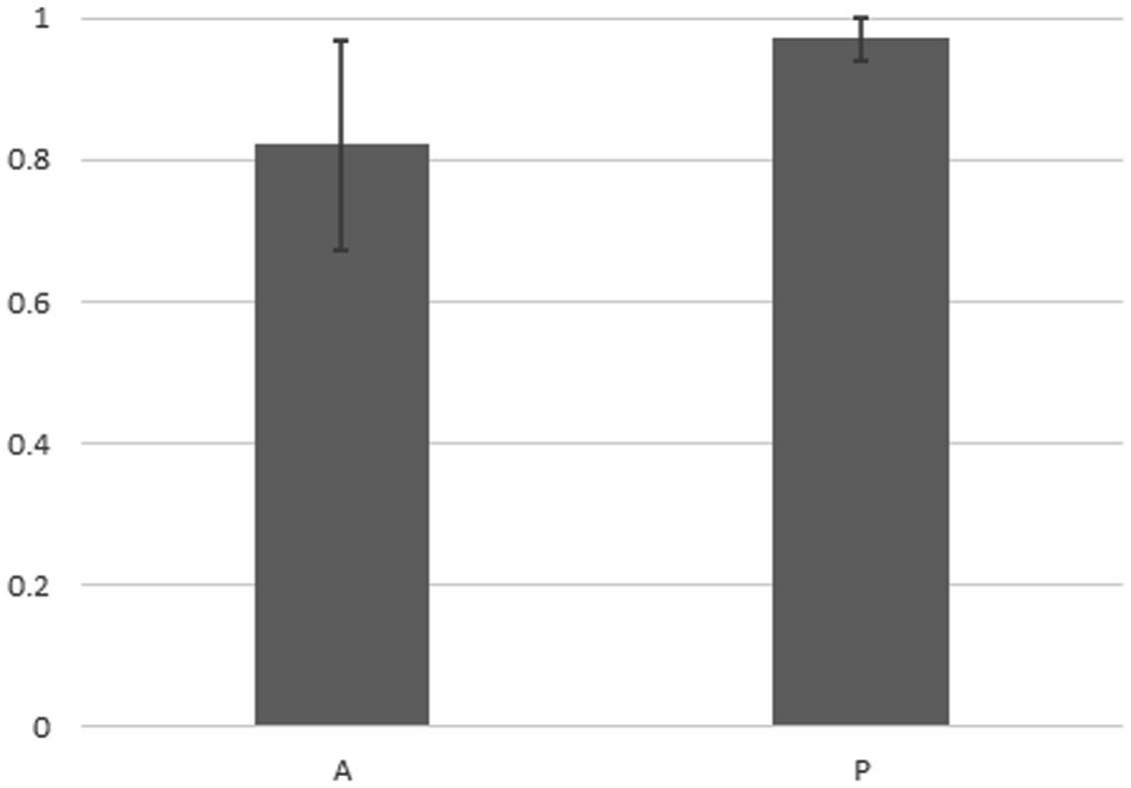
Homology analysis of the microbiome composition between bacteria detected from the unfed *P. bursaria* itself and bacteria detected from the culture medium (LEfSe analysis). The figure shows the mean values of the homology of the eight strains of A (left) and the six strains of P (right) including standard deviation.

**Figure 5:**
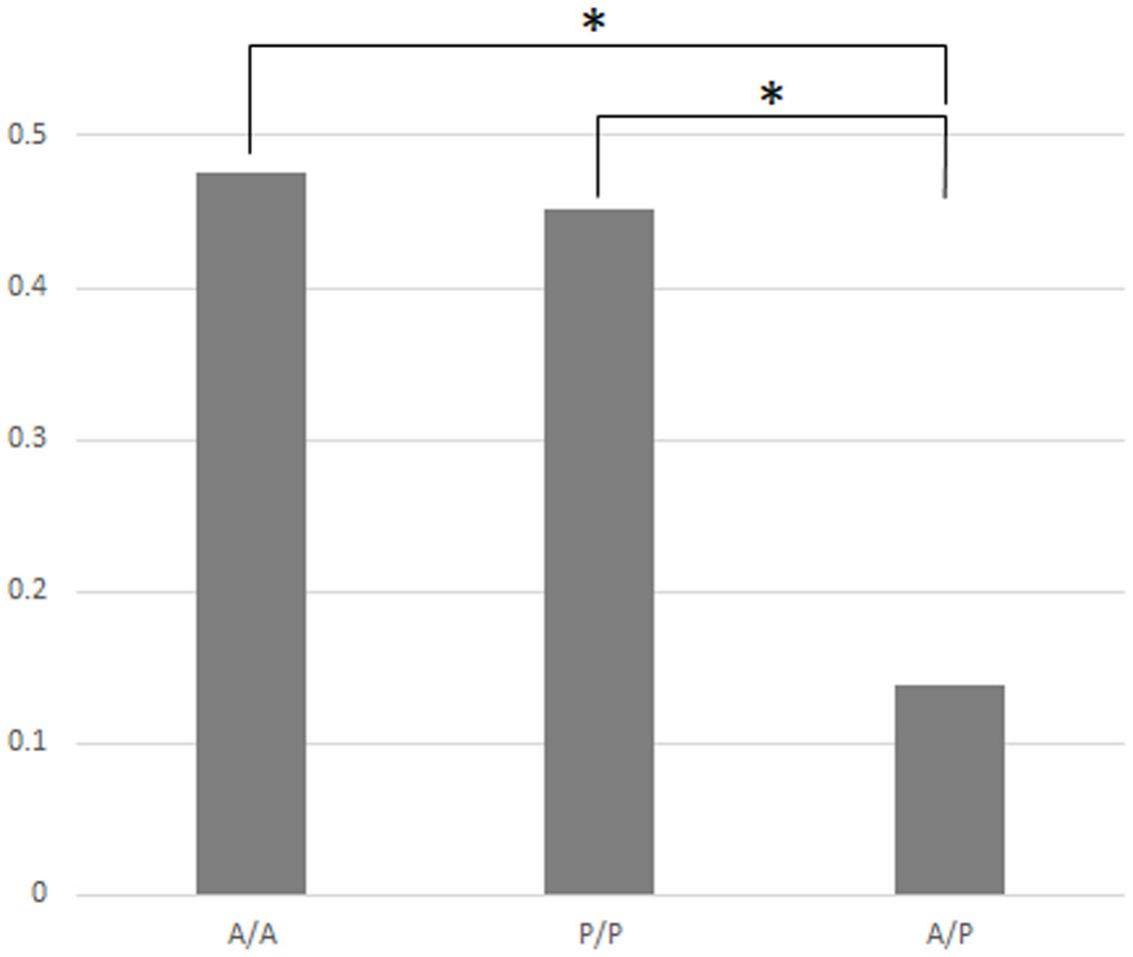
Homology of the bacterial composition among eight actively and six poorly proliferating *P. bursaria* strains, as well as a homology comparison of the microbiome composition between these two types of *P. bursaria* strains (LEfSe analysis). The higher the homology, the closer the numerical value to 1. Left and center bars show mean values compared between the actively proliferating strains (A/A) and poorly proliferating strains (P/P), respectively. The right bar shows the mean of the values compared between these two strains (A/P). Asterisks indicate that differences are statistically significant from each other.

Figure 4 indicates that in the case of the 14 strains shown in Figure 2, the compositions of bacteria detected from the cells of *P. bursaria* and those in the culture medium showed high homology to each other. This was commonly observed in all 14 strains, regardless of whether the proliferation was active or poor. Figure 5 compares the homology of the detected bacterial compositions among the eight strains of *P. bursaria* with active proliferation by LEfSe analysis. A similar homology comparison was also performed among the six strains of *P. bursaria* with poor proliferation. Homology comparisons were also carried out between the former and latter groups, and the obtained values were tallied and compared in Figure 5. There was high homology in bacterial composition among eight strains with active proliferation and six strains with poor proliferation, and the homology in the bacterial composition between the two groups was low.

Furthermore, a 1:1 comparison was made between strains of *P. bursaria* to analyze whether the OTU abundance of particular bacteria differed in both *P. bursaria* strains using differential abundance analysis (Supplementary Table S1). Based on a 1:1 comparison of 14 strains of *P. bursaria*, we found eight types of “bacteria” that tended to differ greatly in abundance between the two strains (dark gray). Among the eight types, the order *Chlorophyta* is most probably 16S RNA gene of the chloroplasts of the algal symbionts of *P. bursaria*. Consequently, we only present the abundance of seven bacteria taxa as summarized in Table 1.

**Table 1:** Comparative analysis of the abundance of bacterial taxa.

	Ratio	Kaistia, 551756	Xanthobacter, 86452	Xanthobacter, 4417061	Variovorax paradoxus, 588664	Variovorax paradoxus, 644798	Sphingomonas, 1024743	Rhodospirillaceae,1640195
A/A	n	16	14	20	7	3	2	14
	% (n/28)	57	50	71	25	11	7	50
P/P	n	8	6	5	13	11	8	3
	% (n/15)	53	40	33	87	73	53	20
A/P	n	28	23	31	27	16	15	21
	% (n/48)	58	48	65	56	33	31	44

For each *P. bursaria* 1:1 combination, the comparative abundance values showing a *p* < 0.05 were indicated in gray in Supplementary Table S1. Next, for each bacterial taxon, the values in gray were counted and designated as n. This *n*-value was then divided by the number of possible 1:1 combinations of *P. bursaria* strains, and bacteria with high values (50% or more, yellow) were selected and summarized. A/A, P/P, and A/P (vertical axis) were compared among the actively proliferating and poorly proliferating *P. bursaria* strains and between the actively and poorly proliferating *P. bursaria* strains, respectively.

For each identified bacterium shown in Supplementary Table S1 (horizontal axis), the abundance of actively proliferating *P. bursaria* strains (eight in red) and poorly proliferating *P. bursaria* strains (six in blue) was further clarified, and the values are summarized in Supplementary Table S2. In 6–7 strains among the eight *P. bursaria* strains with active proliferation, *Kaistia*, *Xanthobacter*, and the family *Rhodospirillaceae* were also abundant. In five strains among the six *P. bursaria* strains with poor proliferation, *Sphingomonas* and *Variovorax* were abundant. The family *Caulobacteraceae* detected in the fed *P. bursaria* strain was also detected in the three unfed strains with active proliferation, but not in all unfed strains with poor proliferation (Table 2).

**Table 2:** Comparison of bacterial taxa detected in fed (2016) and unfed *P. bursaria* (2020) cultures.

Bacteria	2016 fed Pb	2020 unfed Pb		Remarks
	Agar	Microbiome	
			Proliferation
*g_Variovorax*	D	D	P	Root nodule
*g_Caulobacter*	D	(D)	(A)	Cellulolytic
*g_Sphingomonas*	ND	D	P	Bioremediation
*g_Kaistia*	ND	D	A	Microalgae, bioremediation
*f_Rhodospirillaceae*	ND	D		Root nodule, bioremediation
*g_Xanthobacter*	ND	D		Bioremediation

D and ND indicate whether the bacteria were detected or not in a *P. bursaria* strain. A and P indicate if the bacteria were detected in the actively or poorly proliferating *P. bursaria* strains, respectively. On the right, the properties of the bacteria and locations where they have been reported other than those associated with *P. bursaria* are described. Bacterial identification was performed using DNA sequence analysis of bacterial colonies grown on agar plates in 2016, and the microbiome analyzed using 16S rDNA sequencing in 2020. In the genus *Caulobacter* detected only in actively growing *P. bursaria* strains, D and A are shown in parentheses, because it was detected just in 3 out of 8 strains.

## Discussion

Since some bacteria (gram-negative bacilli) can grow and form colonies under the nutritional composition in the BTB agar, while others cannot (see manufacturer’s instructions), the results shown in Figure 1 do not represent all the bacteria that can be identified in an individual *P. bursaria* or media. Nevertheless, three taxa of bacteria were detected in an individual *P. bursaria* after washing with sterile lettuce medium (Figure 1B). It is highly likely that these bacteria adhere to or are in symbiosis with *P. bursaria*. In addition, *Klebsiella* sp. added to the medium in large amounts as food was not detected in *P. bursaria* individuals after washing. Thus, *Klebsiella* sp. was not transferred to symbiosis with *P. bursaria* and was simply digested as food.

As shown in Figure 3, two types of antibiotics with different mechanisms of action, one inhibits bacterial cell wall synthesis (penicillin) and the other one that inhibits bacterial protein synthesis (streptomycin or gentamicin), affected the proliferation of *P. bursaria* cells. It has been reported that several bacteria, including *Escherichia coli*, *Klebsiella* sp., and *Staphylococcus aureus*, are susceptible to penicillin (100 U/mL) /streptomycin (100 μg/mL) or gentamicin (50 μg/mL) (Fischer, 1975). It has also been reported that 20 μg/mL gentamicin is sufficient to inhibit the proliferation of various taxa of bacteria (Kagiwada et al., 2008). As shown in Figure 3B, gentamicin of 10 μg/mL affected the proliferation of *P. bursaria*. This indicates that the presence of bacteria was essential for the proliferation of *P. bursaria*. Previous studies have reported that some protists are sensitive to antibiotics (Chopra and Roberts, 2001; Barnhill et al., 2012), suggesting that antibiotics may affect the mitochondria and plastids of ciliates which might be also possible for *P. bursaria*.

Strains KUHH-3 and KUHH-4 were isolated from strain KUNY-2 in fed culture after washing in 2017 and were continuously cultured in sterile lettuce medium without exogenous feeding. In 2020, we re-isolated and washed these strains and then grew them in sterile lettuce medium to examine their proliferation ability (Supplementary Figure S1). All unfed *P. bursaria* strains had undergone three rounds of cloning after being collected from the pond, so their genetic background was considered to be the same. Nonetheless, the proliferative ability of each individual cell type was extremely diverse.

Although these two strains showed variability in the degree of proliferation, the cell densities in the stationary phase of proliferation of the eight strains in Figure 2 were similar to those in 2017, even after long-term unfed cultivation (see strain KUYY in Figure 3). Based on the data with the KUHH-3 and KUHH-4 strains, we conclude that unfed culture strains of *P. bursaria* have been successfully established. If these unfed strains can be widely shared among researchers of *P. bursaria*, feeding will not be required during culture, and this will greatly contribute to the unification of cultivation conditions among researchers. As a result, it is expected that the reproducibility of the data obtained in the experiments will be significantly improved.

There are numerous possible explanations for the variation in the degree of proliferation between the strains shown in Figure 2. First, it is possible that we were dealing with different sibling species of *P. bursaria* in the original sample. *Paramecium bursaria* has been reported to have five cryptic species worldwide (Greczek-Stachura et al., 2021b; Spanner et al., 2022). However, these cryptic species are presumed to have occurred because of geographic isolation. The unfed *P. bursaria* cells in this experiment showing various proliferation rates were the clonal strain, in which all cells were derived from a single cell obtained from one pond in Japan. Therefore, we think that the possibility that they are cryptic species to each other is extremely low. The second explanation could be the qualitative and/or quantitative diversity of symbiotic algae within the *P. bursaria* cells. Regarding the former, an interesting result was recently reported that different types of symbiotic algae coexisted together with *P. bursaria* (Spanner et al., 2022). This report pointed out that the proliferative activity of *P. bursaria* may differ depending on the type of symbiotic algae in the cell. Regarding the latter, the average content of symbiotic algae in highly proliferative strains might be higher than that in strains with lower proliferation activity. Further studies are required to elucidate the content of symbiotic algae and chlorophyll in the unfed strain of *P. bursaria*. The third explanation could be the diversity of microbes, such as bacteria, other than the symbiotic algae harbored *P. bursaria* cells. Thus far, we believe that this is the most likely explanation.

We showed for the first time that bacteria were still detected in *P. bursaria* cells even when *P. bursaria* was kept for an extended period under “unfed culture” conditions without the addition of exogenous food. The results shown in Figure 4 indicate that the bacterial composition of *P. bursaria* is highly homologous to that present in the culture medium. This suggests that bacteria within *P. bursaria* move in and out of the cell and, in some cases, are taken up as symbionts. It is interesting to note that the genus *Variovorax* detected in the fed strain of *P. bursaria* after collection from the pond (Figure 1) was also detected in the newly established unfed *P. bursaria* strains (Supplementary Tables S1, S2). We therefore think it is highly possible that the genus *Variovorax* is always present in *P. bursaria* cells, and in some cases is symbiotic. The genus *Variovorax* was also abundantly detected in the five *P. bursaria* strains with poor proliferation (Supplementary Table S2). It has also been detected in root nodules of plants and is known as a plant proliferation-promoting bacterium with nitrogen-fixing abilities (Jiang et al., 2012; Satola et al., 2013; Finkel et al., 2020; Perera et al., 2022). It would be interesting if symbiotic algae can escape from *P. bursaria* strains with poor proliferation into the culture medium and receive support such as nitrogen fixation from the genus *Variovorax*. To completely elucidate the role of bacteria in *P. bursaria*, a comprehensive analysis of bacteria should also be performed along with the symbiotic algae isolated from *P. bursaria*.

The genus *Caulobacter* detected in the fed strain (KUNY-2), has cellulolytic activity (Song et al., 2013). It was also detected in actively proliferating unfed *P. bursaria* strains. Having been reclassified into the genus *Sphingomonas*, *Caulobacter* is taxonomically very close to *Sphingomonas* (Chen et al., 2012). From the unfed *P. bursaria* strain, four types of bacteria, the genera *Sphingomonas* (Ye et al., 1996; Asaf et al., 2020), *Kaistia* (Duda et al., 2009; Lee and Jeon, 2018), *Rhodospirillaceae* (Madigan et al., 1984; McEwan et al., 1985) and *Xanthobacte*r (Inguva and Shreve, 1999; Ma et al., 2021) were detected. The genus *Sphingomonas* has been found mainly in poorly proliferating strains, but the other ones in actively proliferating strains (Table 2). It has been clarified that the above-mentioned four types of bacteria exhibit bioremediation activity to decompose organic pollutants and/or nitrogen-fixing activity in plant root nodules. It has also been reported that a bacterium of the family *Rhodospirillaceae* is able to decompose arsenic compounds (Saleem et al., 2019). Here, it is suggested for the first time that the decomposition of pollutants and/or the involvement of nitrogen fixation by these four bacteria might regulate the proliferation of *P. bursaria* and symbiotic algae. Moreover, we detected most probably 16S RNA genes of the chloroplasts of the algal symbionts of *P. bursaria*. Since symbiotic algae might be equally present not only in actively proliferating but also in poorly proliferating strains, chloroplast genes should have been detected equally in both strains. However, as summarized in Table 2, the results for unfed strains in 2020 show that these sequences were abundantly detected from actively proliferating *P. bursaria* strains, contrary to the above expectations. This may be due to the simple reason that, in centrifugation at low speeds, more ciliate cells were recovered in the actively-proliferating strains than in the poorly-proliferating strains, and thus more algal endosymbionts are detected in the former sample.

Several studies have reported the presence of bacteria and/or other microorganisms within ciliates (Görtz, 1982; Gong et al., 2016; Park and Yu, 2018; Mironov and Sabaneyeva, 2020). Alpha- and Gamma-proteobacteria were dominating in bacteria forming consortia with ciliates. Recent 16S rRNA gene metabarcoding analysis revealed that, in *Paramecium* cells, the phylum *Proteobacteria* (including Alpha- and Gamma-Proteobacteria), *Actinobacteria*, and *Firmicutes* were numerous; in *Stentor* cells, *Bacteroidetes*, *Proteobacteria*, and *Firmicutes* were numerous (Lanzoni et al., 2019; Plotnikov et al., 2019). In *P. bursaria*, our data in Supplementary Table S2 indicate that the phyla *Proteobacteria* and *Firmicutes* were the most abundant in 14 unfed strains. Other studies have reported that *Holospora*, *Sonnebornia yantaiensis* and *Francisella novicida* were detected in *P. bursaria* (Rautian et al., 1993; Rautian and Wackerow-Kouzova, 2013; Gong et al., 2014; Watanabe et al., 2022). However, these bacteria were not found in the unfed *P. bursaria* strains established by us.

In the future, it is important to analyze the bacterial communities of the symbiotic algae isolated from *P. bursaria*. We also hope to share the unfed *P. bursaria* strain established in this study with researchers around the world and to promote the unification of cultivation conditions.

## Data availability statement

The data presented in this study are deposited in the repository of the DNA Data Bank of Japan (DDBJ) (DDBJ accession numbers DRA014782 and LC726600-LC726729).

## Author contributions

HH designed the study. EH, TM-A, MK, SK, KI, AH, and HH designed the experiments. EH, IS, SA, YH, GI, YK, KK, DK, AM, YN, YO, SS, NS, KT, HT, YY, KY, NY, and HH conducted the experiments. EH, TM-A, YM, and IS contributed to the bioinformatics analysis. HH and EH wrote the manuscript with contributions from TM-A, YM, IS, SK, KI, and AH. All authors contributed to the article and approved the submitted version.

## Funding

This work was partly supported by grants from the Research Institute for Integrated Science, Kanagawa University (RIIS 201608 to SK, and 201808, 201910, and 202110 to HH), and by Canon Foundation’s research grant program “Creation of Industrial Infrastructure” to HH.

## Conflict of interest

The authors declare that the research was conducted in the absence of any commercial or financial relationships that could be construed as a potential conflict of interest.

## Publisher’s note

All claims expressed in this article are solely those of the authors and do not necessarily represent those of their affiliated organizations, or those of the publisher, the editors and the reviewers. Any product that may be evaluated in this article, or claim that may be made by its manufacturer, is not guaranteed or endorsed by the publisher.

## Footnotes

^1^https://support.illumina.com/documents/documentation/chemistry_documentation/16s/16s-metagenomic-library-prep-guide-15044223-b.pdf

^2^https://doi.org/10.1038/s41587-019-0209-9

^3^https://huttenhower.sph.harvard.edu/lefse/

## Supplementary material

SUPPLEMENTARY TABLE S1 Differential abundance analysis of bacterial taxa detected in *P. bursaria* For each bacterial taxon detected in *P. bursaria* individuals, a Bonferroni correction was performed, and abundances were compared among eight actively proliferating (red) and six poorly proliferating (blue) *P. bursaria* strains using the differential abundance analysis method. The red column indicates the results of a 1:1 comparison (28 combinations in total) among the eight strains. The abundance of each bacterial colony for each combination is shown. The closer this number (p-value) is to 0, the more the abundance of the detected bacteria differ from each other. In the blue column, similar comparisons were made for combinations of six poorly growing strains (15 combinations in total). The red and blue columns are the results of a 1:1 comparison (48 possible combinations in total) between actively and poorly growing strains.

SUPPLEMENTARY TABLE S2 Abundance of bacterial taxa detected in actively and poorly proliferating *P. bursaria* strains. The actively proliferating *P. bursaria* strains (eight in red) and the poorly proliferating *P. bursaria* strains (six in blue) are indicated in Supplementary Table S1. Identified DNA sequences are shown on the righthand side.

SUPPLEMENTARY FIGURE S1 Proliferation curves of *P. bursaria* isolated from long-term unfed cultures (KUHH-3 and 4) on three (October, November and December 2020; **A**, **B** and **D**) and two (November and December 2020; **C** and **E**) occasions, respectively. Statistical analysis was performed using the Kruskal–Wallis test, and each combination was significantly different (*p*<0.05). Abbreviations are as follows, e.g. “liK3a”: “1” represents the data obtained from the first proliferation curve measurement in October, “iK” is the name of the person in charge, and “3” is the KUHH-3 strain. In addition, each person in charge isolated two strains for each proliferation experiment and cultured them separately; the final “a” and “b” represent the first and second proliferation experiment, respectively.
